# The Role of Surgical Techniques in Reducing Postoperative Pain in Abdominal Surgery: Evidence From the PROSPECT Systematic Reviews

**DOI:** 10.1002/wjs.70091

**Published:** 2025-09-13

**Authors:** Stephan Maximilian Freys, Esther Miriam Pogatzki‐Zahn, Narinder Rawal, Girish Premji Joshi, the PROSPECT Working Group of the European Society of Regional Anesthesia and Pain Therapy (ESRA)

**Affiliations:** ^1^ Department of Surgery DIAKO Ev. Diakonie‐Krankenhaus Bremen Germany; ^2^ Department of Anaesthesiology Intensive Care, and Pain Medicine University Hospital Münster Münster Germany; ^3^ Department of Anaesthesiology Orebro University Orebro Sweden; ^4^ Department of Anaesthesiology and Pain Management University of Texas Southwestern Medical Center Dallas Texas USA

**Keywords:** laparoscopic surgery, postoperative pain, procedure‐specific pain management, PROSPECT, surgical technique

## Abstract

**Introduction:**

Inadequate postoperative pain control remains a significant concern as patients continue to experience moderate‐to‐severe pain after surgery. The influence of surgical techniques has not previously been systematically reviewed.

**Methods:**

In this clinical review, data regarding the influence of surgical techniques on postoperative pain after abdominal surgery were extracted and collated from seven published PROSPECT recommendations. The effects of surgical incision and its technique, laparoscopic technique, and local anesthetic infiltration were critically reviewed. The surgical techniques that are recommended and those that are not recommended for the seven procedures are presented.

**Results:**

In general, very few studies have assessed the influence of surgical techniques on postoperative pain management. PROSPECT recommends transverse over vertical incision for open colorectal surgery, and cutting diathermy over use of scalpel in appendicectomy, inguinal hernia, and open colorectal surgeries. For laparoscopic cholecystectomy, a three‐port (vs. four‐port) technique and low‐pressure pneumoperitoneum (< 12 mmHg) are recommended to reduce the risk of postoperative shoulder pain.

**Conclusion:**

This clinical review demonstrates the important role of surgeons in achieving clinically significant benefits by using appropriate incision techniques, laparoscopic methods, and local anesthetic infiltration. Effective pain relief after surgery can improve recovery and reduce the risk of chronic pain. Our study emphasizes the need for future research in this field.

## Graphical abstract

The purpose of this review was to collate evidence from seven existing PROSPECT recommendations: (1) focusing on the impact of various surgical techniques as an important part of procedure‐specific postoperative pain management in abdominal surgery and (2) serving as a practical guidance for the acting surgeon.

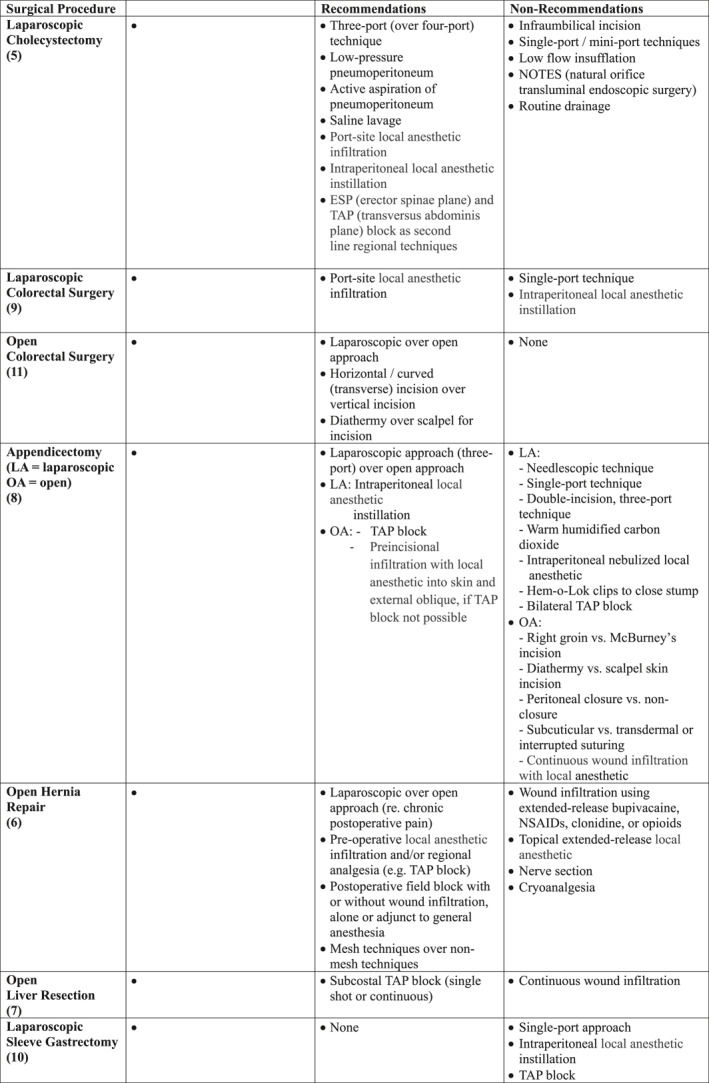

**Abbreviations**
AE: appendicectomy
COX: cyclooxygenase
ESP: erector spinae
IPLA: intraperitoneal instillation of local anesthetics
LCE: laparoscopic cholecystectomy
LCS: laparoscopic colorectal surgery
LSG: laparoscopic sleeve gastrectomy
NSAID: nonsteroidal anti‐inflammatory drugs
OCS: open colorectal surgery
OHR: open hernia repair
OLR: open liver resection
RCT: randomized controlled trial
TAP: transversus abdominis plane

## 1 Introduction

Despite modern clinical and scientific achievements, pain after surgery is not yet optimally managed [1]. Inadequate postoperative pain control remains a significant concern as patients continue to experience moderate‐to‐severe pain after surgery. Optimal pain management is essential for enhanced postoperative recovery and improvement of patient satisfaction [2]. Surgical techniques can play a crucial role in mitigating postoperative pain. Although many guidelines exist for the optimal use of systemic, regional, and nonpharmaceutical analgesic interventions in acute pain management, the impact of surgical techniques on postoperative pain has generally not been assessed. One reason may be that the role of surgical techniques differs between subspecialties. Nevertheless, the identification of procedure‐specific pain management strategies, including surgical techniques, should contribute to improved postoperative pain control.

The procedure‐specific postoperative pain management (PROSPECT) working group of anesthesiologists and surgeons operates under the umbrella of the European Society of Regional Anesthesia and Pain Therapy (ESRA). PROSPECT has been developing practical, procedure‐specific pain management recommendations for over 20 years. These recommendations are derived from the systematic review and critical analysis of available procedure‐specific evidence, supplemented by clinical judgment and a Delphi consensus process. The detailed PROSPECT methodology was first published in 2019 [3] and updated in 2023 [4]. Over the years, more than 25 different surgical procedure‐specific recommendations have been developed, each of them published on the PROSPECT website (https://esraeurope.org/prospect/).

The PROSPECT initiative aims to provide healthcare professionals with practical recommendations for pain management in common but potentially painful operations. The recommendations for each intervention are formulated after a careful evaluation of

- Procedure‐specific evidence, based on a systematic review of the literature.
- The risks and benefits of the intervention in the specific surgical setting.
- Use of the intervention in the context of multimodal, nonopioid and opioid analgesic strategies and modern perioperative care pathways.

These recommendations are updated on the PROSPECT website on a regular basis, offering

- Easily accessible, practical, and pragmatic advice on pain management throughout the perioperative period.
- Evidence‐based arguments for and against the use of analgesic interventions in specific surgical procedures.
- Translations of the summary recommendations in several languages.

The PROSPECT working group meets face to face twice a year, with other communications performed via email correspondence.

The purpose of this clinical review was to collate procedure‐specific evidence for surgical techniques that influence postoperative pain after abdominal visceral surgery, from existing PROSPECT recommendations. This review thus represents a synthesis of seven previously published PROSPECT recommendations, with the focus on the potential role of the surgeon in postoperative pain management. This highlights the important contribution of surgical techniques to pain management. Surgical interventions that could not be recommended due to lack of evidence are also discussed and the need for future investigation of these surgical techniques is explored. The strength of this clinical review lies in the views of an interdisciplinary team of surgeons and anesthesiologists.

## 2 Methods

This clinical review focused on seven published PROSPECT recommendations for abdominal surgery procedures [5, 6, 7, 8, 9, 10, 11] from which data regarding the effects of surgical techniques on postoperative pain were extracted and collated. The recommendations were based on the same methodology [3, 4]. All were published between January 2018 and December 2024, and all are available on the PROSPECT website.

Table 1 presents the PROSPECT methodology for the underlying recommendations, which included

- A systematic review of the literature according to the Preferred Reporting Items for Systematic reviews and Meta‐Analyses (PRISMA) guidelines. A comprehensive search of several electronic databases, including EMBASE, MEDLINE, PubMed, and Cochrane Databases, was performed using appropriate search strings to identify relevant studies. Included studies were randomized controlled trials (RCTs) and systematic reviews or meta‐analyses evaluating the effects of pharmacologic, nonpharmacologic, anesthetic, and surgical interventions reporting pain intensity scores.
- A literature review with study selection using the Cochrane approach.
- Quality assessment of selected studies to assign the level of evidence.
- A critical evaluation for current clinical relevance.
- Assessment of the clinical relevance of differences in pain outcomes (interpreted according to the minimal clinically important difference in pain scores).
- Evaluation of the balance between the efficacy and adverse effect profile of the intervention.
- Assessment of the balance between the invasiveness of the intervention and the degree and consequences of postoperative pain.
- Formulation of recommendations based on the identified evidence; a recommendation for an intervention was only made if at least two positive randomized controlled trials are available supporting improvement of postsurgical pain. Nonrecommendations were made for reasons such as limited, inconsistent, or lack of procedure‐specific evidence.
- A modified Delphi process, which included several rounds of individual comments followed by round‐table discussions to achieve consensus.

**TABLE 1:** PROSPECT methodology for the underlying recommendations.

Surgical procedure	Literature search	PROSPECT methodology	Quality assessment	Anesthesiologists and surgeons	Important limitations
Laparoscopic cholecystectomy [5]	157 RCTs and 31 systematic reviews: August 2017–December 2022	2019 [3]	Modified Delphi version 2 Cochrane risk‐of‐bias tool	+	
Laparoscopic colorectal surgery [9]	72 RCTs: before 25 January 2022	2019 [3]	Modified Delphi	+	Heterogeneous nature of evidence
Open colorectal surgery [11]	13 RCTs: January 2016–January 2022	2023 [4]	Modified Delphi	+	
Appendicectomy (LA = laparoscopic OA = open) [8]	94 RCTs: January 1999–October 2022	2019 [3]	Modified Delphi CONSORT guidelines version 2 Cochrane risk‐of‐bias tool	+	
Open hernia repair [6]	37 RCTs: January 2009–August 2019	2019 [3]	Modified Delphi grading level of evidence	+	Heterogeneous nature of evidence, small size of studies
Open liver resection [7]	31 RCTs and 3 systematic reviews: January 2010–October 2019	2019 [3]	Modified Delphi	+	Heterogeneous nature of evidence, small size of studies
Laparoscopic sleeve gastrectomy [10]	18 RCTs: before September 2018	2019 [3]	Modified Delphi CONSORT guidelines Jadad grading allocation concealment assessment domain 3: rigor of development’ of the AGREE II instrument	+	Heterogeneous nature of evidence, small size of studies, lack of the basic analgesic regimen group

In addition to examining surgical techniques that were or were not recommended within the seven included PROSPECT recommendations, this clinical review explored surgical techniques that have potential for reducing postoperative pain, but were not appropriately evaluated, to provide insights for future studies.

## 3 Results

Seven PROSPECT recommendations on abdominal surgery procedures were included. The procedures are laparoscopic cholecystectomy (LCE) [5], laparoscopic colorectal surgery (LCS) [9], open colorectal surgery (OCS) [11], appendicectomy (AE) [8], open hernia repair (OHR) [6], open liver resection (OLR) [7], and laparoscopic sleeve gastrectomy (LSG) [10].

The recommendations and nonrecommendations for each surgical procedure are presented in Table 2, focusing on those related to specific surgical techniques: surgical incision, incision technique, laparoscopy techniques, and use of local/regional anesthetic techniques.

**TABLE 2:** Effects of surgical techniques on postoperative pain: evidence from PROSPECT systematic reviews.

Surgical procedure	Recommendations	Non‐recommendations
Laparoscopic cholecystectomy [5]	Three‐port (over four‐port) technique Low‐pressure pneumoperitoneum Active aspiration of pneumoperitoneum Saline lavage Port‐site local anesthetic infiltration Intraperitoneal local anesthetic instillation ESP (erector spinae plane) and TAP (transversus abdominis plane) block as second line regional techniques	Infraumbilical incision Single‐port/mini‐port techniques Low flow insufflation NOTES (natural orifice transluminal endoscopic surgery) Routine drainage
Laparoscopic colorectal surgery [9]	Port‐site local anesthetic infiltration	Single‐port technique Intraperitoneal local anesthetic instillation
Open colorectal surgery [11]	Laparoscopic over open approach Horizontal/curved (transverse) incision over vertical incision Diathermy over scalpel for incision	None
Appendicectomy (LA = laparoscopic OA = open) [8]	Laparoscopic approach (three‐port) over open approach LA: Intraperitoneal local anesthetic instillation OA: — TAP block — Preincisional infiltration with local anesthetic into skin and external oblique, if TAP block not possible	LA: — Needlescopic technique — Single‐port technique — Double‐incision, three‐port technique — Warm humidified carbon dioxide — Intraperitoneal nebulized local anesthetic — Hem‐o‐Lok clips to close stump — Bilateral TAP block OA: — Right groin versus McBurney's incision — Diathermy versus scalpel skin incision — Peritoneal closure versus nonclosure — Subcuticular versus transdermal or interrupted suturing — Continuous wound infiltration with local anesthetic
Open hernia repair	Laparoscopic over open approach (re. Chronic postoperative pain) Preoperative local anesthetic infiltration and/or regional analgesia (e.g., TAP block) Postoperative field block with or without wound infiltration, alone or adjunct to general anesthesia Mesh techniques over nonmesh techniques	Wound infiltration using extended‐release bupivacaine, NSAIDs, clonidine, or opioids Topical extended‐release local anesthetic Nerve section Cryoanalgesia
Open liver resection [7]	Subcostal TAP block (single shot or continuous)	Continuous wound infiltration
Laparoscopic sleeve gastrectomy [10]	None	Single‐port approach Intraperitoneal local anesthetic instillation TAP block

## 4 Discussion

The sequence of technical measures taken during an operative procedure is often ruled by tradition. Many everyday decisions follow such traditions without questioning them. This clinical review was inspired by a desire to look into the details, with curiosity about the technical steps of the operative process and the procedure‐specific evidence behind them. The recommended steps are simple measures in any perioperative process that may well be exercised by the responsible surgeon.

In many cases, the choice of surgical technique is based upon reasons other than postoperative pain; a complex interplay of several factors such as the characteristics of surgical pathology, technical feasibility, surgeon's expertise, and availability of local resources as well as patient‐related factors such as comorbid conditions and patient choice (shared decision‐making).

However, surgical techniques can significantly influence postoperative pain outcomes. An important finding is the fact that specific surgical techniques (e.g., port site local anesthetic infiltration, transversus abdominis plane (TAP) block, intraperitoneal local anesthetic instillation) have a clinically and statistically significant impact in certain procedures, whereas the evidence does not support this effect in other procedures. Not all of the examined surgical techniques are applicable in every operative procedure. Indeed, the underlying seven PROSPECT recommendations show a rather diverse picture with regards to the representation of the different surgical techniques. Differences between the recommendations may be due to the evidence base:

- Recommended techniques must be supported by sufficient evidence to be accepted by the PROSPECT methodology (a recommendation is only made if at least two positive RCTs are available).
- Some techniques were studied but there is insufficient supporting evidence to recommend them according to the PROSPECT methodology in certain operative procedures.
- Other techniques were not applied in all operative procedures.

This review leads to two key findings: firstly, few studies have assessed the impact of surgical techniques on postoperative pain management for abdominal surgery; secondly, the role of the surgeon in influencing postoperative pain is emphasized by a clinically significant, positive effect of a horizontal/curved surgical incision in OCS, as well as benefits of laparoscopy over open surgery, if possible, and of certain local/regional anesthetic techniques in specific procedures.

## 5 Surgical Incision

For OCS, PROSPECT recommends a horizontal/curved (transverse) incision over a vertical incision [11]. There is an ongoing discussion, quite often stimulated by members of different “surgical schools”, whether to use midline or transverse incisions for conventional abdominal operations. Most comparative studies concerning this question focus on the problem of hernia formation rather than on postoperative pain. A quite old systemic review states that both analgesic use and pulmonary compromise may be reduced with a transverse or oblique incision. The authors emphasize methodological and clinical diversity and the potential for bias in the included studies and thus the conclusions of this study should be treated with caution. Despite these limitations, the authors suggest that the type of incision for abdominal surgery should remain the preference of the surgeon [12]. This is not in disagreement with the PROSPECT recommendation on OCS; if a vertical incision is required for surgical reasons, it should be done. However, if a horizontal/curved incision is possible, this should be preferred to avoid pain. In addition, transverse or curved incisions will make the use of some regional techniques more effective or easier, since fewer dermatomes are involved compared to vertical techniques.

Importantly, a generalization toward other abdominal operations is not possible, not due to a lack of potential benefit of the horizontal or curved incision but because of the absence of supporting evidence.

## 6 Incision Technique

There are a few studies dealing with incisional techniques and their impact on postoperative pain. In an early, single‐center RCT, reduced postoperative pain scores and total analgesic requirements, as well as improved peak expiratory flow rates, were documented in patients following cutting diathermy versus scalpel incision of the abdominal wall, below the level of the skin, for conventional cholecystectomy [13]. A randomized trial then reported that electrosurgical midline incision in elective surgery had significant advantages over scalpel use on the basis of incision time, blood loss, early postoperative pain, and analgesic requirements [14]. In accordance, a meta‐analysis demonstrated a lower incidence of early postoperative pain, speed of the technique, and reduced blood loss, supporting routine use of cutting diathermy in preference to scalpel use for abdominal skin incisions [15]. Two further meta‐analyses found no differences in the rate of wound complications or postoperative pain between the two techniques [16, 17]. Based on all these studies, the use of cutting diathermy for skin incisions in OCS is recommended by PROSPECT, in preference to the use of a scalpel [11].

## 7 Minimally Invasive Approach

With the advent of laparoscopic surgery, a multitude of publications have focused on its pros and cons. The minimally invasive approach has been recommended because it is associated with a reduced surgical stress response and consequent enhanced recovery. Thus, the traditional open (conventional) approach has been replaced in many abdominal procedures by a laparoscopic approach (e.g., cholecystectomy, colorectal surgery, appendicectomy, antireflux surgery, and bariatric surgery), which has become a standard of care. Also, in turn, the minimally invasive approach led to the development of new operations (e.g., total extraperitoneal hernioplasty for inguinal hernia repair, retroperitoneal adrenalectomy). In addition, this process is being propagated by the introduction of robotic surgery, with further developments of laparoscopic techniques and the invention of new operations, or at least steps of certain operations, using this new technique. To date, there is insufficient (and an urgent need for) evidence on the impact of robotic surgery on postoperative pain according to the PROSPECT methodology.

The use of laparoscopic surgical procedures was associated with less pain than their equivalent open surgical procedures, as reported in three PROSPECT recommendations [6, 8, 11]. However, the use of a laparoscopic approach alone is not necessarily sufficient; if a laparoscopic procedure is used, several additional perioperative measures should be considered by the surgeon to reduce pain.

### 7.1 Number and Size of Trocars

There is an ongoing trend to try to minimize the number and size of trocars (e.g., the use of single‐port techniques) to further reduce the surgical stress response. However, single‐port techniques are not recommended for LCE [5], laparoscopic AE [8], and LCS [9] because of inconsistent evidence of improved pain relief and increased risk of postoperative complications. Similarly, needlescopic (mini‐port) laparoscopic AE and double‐incision, three‐port laparoscopic AE could not be recommended because of a lack of procedure‐specific evidence. However, for LCE, a three‐port technique is recommended over a four‐port technique.

### 7.2 Low‐Pressure Pneumoperitoneum

Although the laparoscopic approach is associated with significantly lower postoperative pain than the conventional open approach, it is associated with severe abdominal discomfort and shoulder pain, probably due to diaphragm irritation caused by CO_2_ [18]. In the recent PROSPECT recommendation on LCE [5], a low‐pressure pneumoperitoneum, defined as pressure below 12 mmHg, is recommended to reduce postoperative shoulder pain. In the other six PROSPECT recommendations, there are insufficient data dealing with this issue and therefore no recommendation could be made. However, this does not mean that for these procedures a minimization of pressure might not help to reduce shoulder pain.

### 7.3 Warmed, Humidified Gas

It has been suggested that warmed and humidified carbon dioxide (CO_2_) used for abdominal insufflation might avoid hypothermia and reduce postoperative pain, especially in long procedures with a high turnover of gas. However, evidence was identified only for AE [8] and that suggests a lack of effect on pain. A comparison of warmed, humidified CO_2_ insufflation with standard insufflation yielded no difference in pain intensity or opioid consumption [8]. In the other six surgical procedures, there were no data available to address this issue. Thus, warmed and/or humidified CO_2_ is not recommended in the relevant PROSPECT recommendations because the evidence for reduced postoperative pain is lacking.

### 7.4 Desufflation

In the LCE recommendation, the literature favors an active aspiration of the pneumoperitoneum, which is easy to perform, because it significantly reduced pain scores, although no difference was found in postoperative opioid consumption [5]. Again, in the literature addressing the six other procedures, there are insufficient data dealing with this issue.

## 8 Local/Regional Anesthetic Techniques

Surgeon‐administered local/regional analgesic techniques are increasingly common [19]. The most frequently practiced techniques before or during surgery are, in order of popularity, a single‐shot surgical site infiltration, interfascial plane blocks, TAP blocks, and a single‐shot intraperitoneal instillation.

### 8.1 Surgical Site Local Anesthetic Infiltration

Surgical site local anesthetic infiltration is a simple, low‐cost approach to reducing postoperative pain. However, the evidence of its benefits is conflicting, most likely due to an inconsistent approach to infiltration. In LCE [5], LCS [9], open AE [8], and OHR [6], a surgical site infiltration is recommended; however, several specific points should be understood:

LCE: Surgical port site infiltration is recommended. Even though no studies were found comparing the timing of port site local anesthetic infiltration before or after incision, the use of preemptive analgesia may result in a decreased surgical stress response and more effective pain control postoperatively. A TAP block offers superior pain relief; however, performing wound infiltration is a much easier technique than a TAP block.

LCS: Surgical port site wound infiltration is recommended despite inconsistent evidence because of its simplicity and low costs, although TAP blocks appear to have superior analgesic effects. Of note, performing surgical site infiltration is easier than performing interfascial plane blocks.

AE: Preoperative local anesthetic infiltration into the skin and deep to the external oblique muscle is recommended in open AE if a TAP block is not possible. Because of a lack of studies on wound infiltration in laparoscopic AE, there was no evidence to formulate recommendations for this type of surgical procedure.

OHR: Local anesthetic infiltration and/or regional analgesia (ilio‐hypogastric/ilio‐inguinal nerve block or TAP block) are recommended. With regards to the choice ^of^ anesthetic technique, the PROSPECT recommendation supports the use of a field block (e.g., ilio‐inguinal/ilio‐hypogastric block) with or without wound infiltration as a sole anesthetic or as an adjunct to general anesthesia.

### 8.2 Interfascial Plane Blocks

Interfascial plane blocks are increasingly used for several surgical procedures, particularly for abdominal surgery. These blocks have replaced neuraxial blocks (e.g., epidural analgesia and paravertebral blocks), which are being phased out [20]. Currently, the most commonly used interfascial plane block is the TAP block. Although these techniques are generally applied by anesthesiologists, surgeons may also perform them. The currently available evidence from six of the seven PROSPECT recommendations paints the following picture [5, 6, 7, 8, 9, 19]:

LCE: Erector spinae plane (ESP) and TAP blocks are recommended. Although TAP blocks have a superior analgesic effect compared to port site infiltration, it is important to consider that port site infiltration is a much simpler procedure to perform.

LCS: Due to an inconsistent procedure‐specific evidence base, truncal blocks are not recommended as first‐line treatment. TAP blocks may possibly offer clinical benefits if an additional incision is created (hand‐assisted surgery).

AE: Open AE: A preoperative unilateral TAP block is recommended. The existing studies revealed a significant and clinically relevant benefit of a TAP block as a component of multimodal analgesia with no increase in complications. There is only limited evidence to add analgesic adjuncts like morphine to prolong a local anesthetic‐based TAP block and so this is not recommended.

Laparoscopic AE: Bilateral TAP block showed promising results, but because of limited procedure‐specific evidence, it is not recommended.

OHR: Local anesthetic infiltration and/or regional analgesia (ilio‐hypogastric/ilio‐inguinal nerve block or TAP block) are recommended. With regards to the choice of anesthetic technique, the PROSPECT recommendation supports the use of a field block (e.g., ilio‐inguinal/ilio‐hypogastric block) with or without wound infiltration as a sole anesthetic or as an adjunct to general anesthesia.

OLR: Interfascial plane blocks (e.g., subcostal TAP, quadratus lumborum, and ESP blocks), administered either as single‐shot injection or as continuous local anesthetic infusion, have been shown to be efficacious. However, due to limited procedure‐specific evidence, the PROSPECT recommendation states that only subcostal TAP blocks should be considered. To cover the entire surgical incision, these TAP blocks should be performed bilaterally and local anesthetic should be distributed in a subcostal and lateral approach. Since there are no studies comparing the continuous infusion of local anesthetic via catheters placed in the TAP space to a single‐shot infiltration, preference cannot be given to one or the other technique.

LSG: Although a number of studies evaluated TAP blocks in LSG, the relevant PROSPECT recommendation concludes that despite the reported analgesic benefits of TAP blocks, port‐site infiltration is recommended over TAP blocks, since the TAP block techniques used varied significantly.

### 8.3 Intraperitoneal Instillation

Intraperitoneal instillation of local anesthetic (IPLA) during laparoscopic surgery has produced conflicting results, most likely due to significant variation in technique, such as local anesthetic dose and volume, the site of instillation, and the timing of instillation. This technique is not applicable and/or has not been mentioned in open procedures such as OCS, OHR, and OLR.

Again, the existing evidence differs from one laparoscopic procedure to another [5, 8, 9, 19]:

LCE: The available evidence underlying the PROSPECT recommendation on LCE demonstrates that IPLA may offer an additional benefit above basic analgesia and port site infiltration. A comparison between IPLA and port site infiltration did not demonstrate a clinically or statistically relevant difference in postoperative pain scores between the two. Therefore, in LCE, both techniques are recommended with no preference. However, a combination of both techniques should be avoided to prevent local anesthetic systemic toxicity (LAST).

AE: Laparoscopic AE: IPLA during laparoscopic AE is recommended although there were methodological differences in the underlying evidence regarding basic analgesia and a reported variability in placement. A clear advantage is seen in the lack of relevant side effects and the simple method of application.

LCS: The underlying evidence for the PROSPECT recommendation for LCS paints a different picture. Although there are several reports on analgesic and opioid‐sparing effects, there were no data in the context of adequate baseline analgesia and the data are too inconsistent to recommend IPLA for LCS. However, IPLA may be used if basic analgesia or intravenous lidocaine cannot be provided.

LSG: The available evidence for LSG did not demonstrate that IPLA improved pain scores and reduced opioid use; thus, no positive recommendation was possible.

## 9 Balanced Analgesia Concept

The concept of balanced analgesia was introduced to improve analgesic efficacy and reduce the dose and adverse effects of drugs like opioids [21, 22]. All PROSPECT recommendations therefore include basic analgesics like acetaminophen and nonsteroidal anti‐inflammatory drugs (NSAIDs) or cyclooxygenase (COX)‐2 specific inhibitors, administered either pre‐ or intraoperatively, and continued postoperatively, unless there are any contraindications. As indicated in the detailed recommendations, these basic analgesics should be supplemented with procedure‐specific local and/or regional analgesia or specific surgical techniques and—for some procedures—with drugs like oral gabapentinoids (preoperatively and only if no basic analgesic is possible for laparoscopic sleeve gastrectomy [2]) or intravenous dexamethasone (e.g., for laparoscopic cholecystectomy [5]). Opioids should be reserved for rescue.

## 10 Limitations

The limitations in this clinical review are related to those of the studies included in the systematic reviews of the seven underlying surgical procedures. Overall, there is considerable heterogeneity between studies. Importantly, many studies did not include basic analgesics (i.e., acetaminophen or NSAIDs or COX‐2 specific inhibitors) as part of a multimodal analgesic regimen. Also, local/regional analgesic techniques were not always used. As PROSPECT methodology has been refined in recent updates, the inclusion or absence of basic analgesia in the underlying studies has become a key factor when considering the clinical relevance of the evidence. As a result, there may be differences between earlier and more recent PROSPECT recommendations in Table 2, in terms of the interpretation of supporting studies and their use of basic analgesia.

## 11 Conclusions

This clinical review examined the impact of various surgical techniques as an important part of procedure‐specific postoperative pain management in abdominal surgery. This includes techniques that minimize tissue trauma, such as the minimally invasive approach, meticulous dissection techniques and nerve preservation, effective hemostasis, local anesthetic surgical site infiltration or intraperitoneal instillation, application of intrafascial plane blocks, management of the pneumoperitoneum, and incision techniques.

The findings emphasize the importance of reviewing the details of any surgical procedure when adopting advanced surgical techniques and integrating procedure‐specific postoperative pain management into surgical practice.

Finally, it should be clearly understood that applying such techniques can make a remarkable difference for individual patients, regardless of whether they are supported by scientific evidence, as expressed in the PROSPECT recommendations, or by clinical experience, or even by common sense.

## Author Contributions

**Stephan Maximilian Freys:** project administration, supervision, conceptualization, methodology, validation, writing – original draft, writing – review and editing. **Esther Miriam Pogatzki‐Zahn:** conceptualization, methodology, validation, writing – original draft, writing – review and editing. **Narinder Rawal:** conceptualization, methodology, validation, writing – original draft, writing – review and editing. **Girish Premji Joshi:** conceptualization, methodology, validation, writing – original draft, writing – review and editing.

## Conflicts of Interest

The PROSPECT working group is supported by an unrestricted grant from the European Society of Regional Anesthesia and Pain Therapy (ESRA). In the past, PROSPECT has received unrestricted grants from Pfizer Inc. New York, New York, USA and Grünenthal Germany.

Stephan Maximilian Freys received financial support for advisory and lecture fees from Grünenthal, Germany, Pajunk Germany and Ever Pharma Germany. All money went to the institution (DIAKO Bremen) he is working for.

Esther Miriam Pogatzki‐Zahn received financial support from Grünenthal Germany for research activities and advisory and lecture fees from Grünenthal Germany, Novartis Germany, and Medtronic Germany. In addition, she receives scientific support from the German Research Foundation (DFG), the Federal Ministry of Education and Research (BMBF), the Federal Joint Committee (G‐BA), and the Innovative Medicines Initiative 2 Joint Undertaking under grant agreement no. 777500. This Joint Undertaking receives support from the European Union's Horizon 2020 research and innovation program and EFPIA. All money went to the institutions (WWU/UKM) she is working for.

Narinder Rawal has no conflicts of interest.

Girish Premji Joshi received honoraria for consultation from Vertex Pharmaceuticals and Haidco‐USA Pharmaceuticals.

## Data Availability

The data that support the findings of this study are available from the corresponding author upon reasonable request.
